# Exploring Stratification Strategies for Population‐ Versus Randomization‐Based Inference

**DOI:** 10.1002/pst.2419

**Published:** 2024-07-10

**Authors:** Marco Novelli, William F. Rosenberger

**Affiliations:** ^1^ Department of Statistics Bologna University of Bologna Bologna Italy; ^2^ Department of Statistics George Mason University Fairfax Virginia USA

**Keywords:** chronological bias, poststratification, prestratification, randomization tests, regression adjustment, subgroup analysis

## Abstract

Stratification on important variables is a common practice in clinical trials, since ensuring cosmetic balance on known baseline covariates is often deemed to be a crucial requirement for the credibility of the experimental results. However, the actual benefits of stratification are still debated in the literature. Other authors have shown that it does not improve efficiency in large samples and improves it only negligibly in smaller samples. This paper investigates different subgroup analysis strategies, with a particular focus on the potential benefits in terms of inferential precision of prestratification versus both poststratification and post hoc regression adjustment. For each of these approaches, the pros and cons of population‐based versus randomization‐based inference are discussed. The effects of the presence of a treatment‐by‐covariate interaction and the variability in the patient responses are also taken into account. Our results show that, in general, prestratifying does not provide substantial benefit. On the contrary, it may be deleterious, in particular for randomization‐based procedures in the presence of a chronological bias. Even when there is treatment‐by‐covariate interaction, prestratification may backfire by considerably reducing the inferential precision.

## 1 Introduction

In this paper, different stratification strategies from the perspective of both population and randomization‐based inference are investigated. There are numerous approaches in population‐based inference aimed at taking into account the information provided by the baseline covariates: prestratification, poststratification, and regression modeling. Most clinical trials prestratify on certain important variables, such as clinic, gender, or age, since ensuring cosmetic balance on known baseline covariates is often deemed to be a crucial requirement for the credibility of the trial results [1, 2]. The general philosophy (although not always practiced in the medical literature) is that a stratified analysis demands a stratified test. But a stratified test can be conducted whether the trial was prestratified (called poststratification). Another approach makes use of regression models to adjust the treatment effect analysis post hoc, whether the trial was prestratified.

Randomization‐based inference has analogous testing procedures. However, prestratification allows analysis of separate strata, or elimination of strata, due to the fact that a separate randomization procedure was used within each strata, so removing a stratum does not affect the randomization distribution. Poststratification can be accomplished using rerandomization by fixing the strata and responses and rerandomizing according to the unstratified randomization procedure. The test statistic is computed in each stratum and the tests are combined (weighted, if desired), into a single stratified test. Finally, a regression analysis can be used by permuting the residuals of any regression model that does not include a treatment effect (under the null hypothesis), as shown in Gail, Tan, and Piantadosi [3], and Parhat, Rosenberger, and Diao [4]. Some might naturally ask why a model cannot be fitted that includes treatment, then compute the estimated covariate‐adjusted treatment effect and use that as the metric for the randomization. Philosophically, fitting such a model does not assume the null hypothesis that the treatment effect is zero, but the idea is consistent with the way we conduct parametric model‐fitting. One might intuit that a test on the residuals would be more robust to model misspecification than a test on the fitted model parameters.

Whether to stratify on important known covariates has been debated in the literature for many years and has not yet been resolved. It is likely that agreement has been reached on two issues: (i) a stratified analysis should follow a stratified trial and (ii) a poststratification via a stratified test or regression model can be done whether or not the trial was stratified on those covariates. Beyond this, the literature has shown that prestratification does not improve efficiency in large samples and improves it only negligibly in smaller samples [5]. Ganju and Zhou [6] show that, employing permuted block randomization, prestratification can actually have a negative impact if there is a treatment‐by‐covariate interaction, so that there are differential treatment effects across subgroups. Only in the event that the stratum mean square error is much larger than the overall mean square error with the interaction term does prestratification seem to be more efficient. Indeed, while prestratifying on baseline covariates can mitigate accidental bias, by inducing independence between the treatment effect and unobserved covariates, it has no impact on the presence of treatment‐by‐covariate interactions since these are true features of the phenomena under study and not the result of chance [1]. As Permutt highlighted, it is principally the stratified analysis that can both eliminate bias and identify heterogeneity among subgroups, while the stratified randomization is often less important than believed [7].

Numerous studies have thoroughly examined this topic from various perspectives. Li and Ding [8] discussed the benefits of combining rerandomization in the design stage with regression adjustment demonstrating that combining these two methods improves statistical inference. In the case of completely randomized experiments, Liu and Yang [9] analyzed the regression‐adjusted average treatment effect compared to the stratified difference in means estimator. Their analysis revealed that the former generally has a smaller asymptotic variance than the latter. Shao, Yu, and Zhong [10] provided some theoretical results for testing hypotheses under covariate‐adaptive randomization along with a valid bootstrap *t*‐test, which is exact in the sense that its limiting rejection probability under the null hypothesis is equal to the nominal level. Additionally, Bugni, Canay, and Shaikh [11] addressed the issue of inference for the average treatment effect in covariate‐adaptive designs, later generalized to multiple treatments [12]. In the case of two competing treatments, they examined the behavior of the unstratified two‐sample *t*‐test, its fixed effects regression‐adjusted version, and its permutation version. The authors demonstrated that the unstratified two‐sample *t*‐test is generally conservative. However, they found that both the regression‐adjusted and permutation versions maintain the type I error rates when the “target” proportion of units assigned to treatment in each stratum is balanced.

One can consider this paper the logical extension of early work by Matts and McHugh [13] and Davis [14] that explored the properties of prestratified and poststratified randomization tests asymptotically. In those days, the rerandomization test would have been difficult or impossible computationally, and there is considerable complexity in determining the randomization distribution of a stratified test in the event that the trial was not prestratified. Nowadays, we do not have these difficulties. Our study aims to contribute to the existing literature by simultaneously examining the effects of pre‐ versus poststratification, as well as population‐ versus randomization‐based inference, comparing several restricted randomization procedures. To the best of our knowledge, this is the first attempt to provide a comprehensive comparison of the operating characteristics of various restricted randomization procedures within two inferential frameworks for distinct stratification strategies. Moreover, the impact of the presence of both treatment‐by‐covariate interaction and variability in patient responses on the inferential accuracy is investigated. In particular, in what follows three main questions about the potential benefit of prestratification will be addressed.

1. Is prestratification beneficial in terms of inferential precision? If so, what are the pros and cons of population‐based versus randomization‐based stratified analyses?
2. In the regression‐adjustment approach, what is the best way to exploit randomization‐based inference? Namely, does permuting the treatment effect provide some benefit over the rerandomization of residuals?
3. How does the presence of a treatment‐by‐covariate interaction affect the previous results? Is prestratification beneficial when there is heterogeneity among subgroups?

One of the main aims of this work is to compare the performances of the two inferential techniques in analyzing randomized clinical trials. Randomization‐based inference has long been heralded as an appropriate technique to analyze randomized experiments, going back to Fisherian times. Since randomized clinical trials do not involve sampling from a population, applying inference techniques that derive their philosophical and theoretical basis from the random sampling mechanism are, at best, approximate, and, at worst, inappropriate. As Cornfield [15] noted, randomization itself makes possible the answer to the question “In how many experiments could a difference of this magnitude have arisen by chance alone if the treatment truly has no effect?” The implication is that the answer to this question is not possible except by *replication* of the experiment, unless randomization is employed. Kempthorne [16] provides another benefit of randomization‐based inference (talking about his 1952 book):

… if one has randomized and one considers the data in the randomization frame, then the probability that the significance level (called *p*‐value by some) is less than or equal to α is, in fact, equal to α. It is sort of a concomitant of the randomization. The significance levels, given by randomization tests are, so to speak, believable.

The preservation of type I error rates in a randomized clinical trial has become sacrosanct in the regulatory agencies and among the multiple testing and sequential monitoring biostatistics community. And yet rarely are randomization‐based inference techniques actually used following randomized clinical trials. Instead many biostatisticians rely on population‐based tests that may, under certain parametric or asymptotic assumptions, preserve type I error rates. Folks [16] gets to the heart of the matter of why they were not used in Kempthorne's day, in his conversation with Kempthorne: “… randomization tests then were not possible. One didn't do them because of the computation.” Nowadays, rerandomization tests computed by generating a large number of randomization sequences and recomputing the test statistic for each can be done in seconds [1, 17]. A simple proof of the preservation of type I error rates at the nominal level for rerandomization tests is found in Prochan and Dodd [18].

One criticism of randomization‐based inference is that it primarily focuses on the simple null hypothesis of no treatment effect. Indeed, a crucial distinction between the two inferential approaches pertains to the hypothesis being tested. Differently from the usual parametric poluation‐based approach, under the null hypothesis of no treatment effect, the randomization‐based one posits that patient responses remain unaffected by either of the two assigned treatments. This hypothesis does not involve any parameters and essentially asserts that the assignment of treatments is independent of the outcomes for patients [1, 19]. In Section 4.2, this issue is further explored by also comparing the performances of the two inferential approaches under “strong” and “weak” null hypothesis [20]. However, it is not difficult to extend randomization‐based inference to comparisons of multiple treatments [21], confidence interval estimation [21], covariate‐adjusted regression models [4], and sequentially monitored outcomes [22]. In fact, every type of primary outcome analysis that is standardly employed using population‐model inference techniques can be accomplished using a rerandomization test.

The paper is organized as follows. Starting from some preliminaries in Section 2, Section 3 answers question (1) by discussing pros and cons of prestratification combined with the comparison of population versus randomization‐based inference. Section 4 is dedicated to answering question (2) by examining the regression‐based adjustment approaches. Question (3) instead is addressed in the previous two sections to highlight if and how the presence of treatment‐by‐covariate interaction interplays with the stratification approach adopted. Finally, Section 5 presents some concluding remarks.

## 2 Notation/Background

Consider a randomized clinical trial in which patients arrive sequentially and are assigned to one of two competing treatments, say A and B. Let n be the total number of patients to be enrolled and $\delta_{i}\;\left(i=1,,,\ldots ,,,n\right)$ the treatment indicator equal to 1 if the i‐th patient is assigned to A and 0 if B. Suppose that for each patient a qualitative covariate Z with K strata is observed; then $y_{\mathrm{ikj}}$, the response of the patient i in stratum k assigned to treatment $j\;\left(\;j=A,B\right)$ is assumed to follow the linear model

$$y_{\mathrm{ikj}}=\mu +\beta_{j}+z_{k}+{\left(\mathrm{\beta z}\right)}_{\mathrm{kj}}+\epsilon_{i}$$

where μ is the overall mean, $\beta_{j}$ the effect of treatment j, $z_{k}$ the covariate effect in stratum k, and ${\left(\mathrm{\beta z}\right)}_{\mathrm{kj}}$ the treatment‐by‐covariate interaction, namely the effect of treatment j within stratum k; finally, $\epsilon_{i}$ is the error component that, unless otherwise stated, is assumed to follow a standard normal distribution. Furthermore, let Δ be the K‐dimensional vector collecting $\Delta_{k}$, the treatment effect in stratum k, namely

$$\Delta_{k}=\mu_{\mathrm{kA}}-\mu_{\mathrm{kB}}=\beta_{A}-\beta_{B}+{\left(\mathrm{\beta z}\right)}_{\mathrm{kA}}-{\left(\mathrm{\beta z}\right)}_{\mathrm{kB}}\;k=1\ldots K$$

where $\mu_{\mathrm{kA}}$ and $\mu_{\mathrm{kB}}$ are the population mean responses in stratum k for treatment *A* and *B*, respectively.

In what follows, trials with n=100 subjects and K=4 strata will be considered. Results for n=50 and 200 are provided in Appendix A. Five randomization procedures will be compared: Efron's biased coin design (BCD) with biasing probability set equal to 2/3, the big stick design (BSD) with maximum tolerated imbalance equal to 3, the permuted block design (PBD) with blocks of size 6, the random block design (RBD) with blocks of size 4, 6, or 8 and the complete randomization (CR)—see Chapter 3 of Rosenberger and Lachin [1] for details. The presence of variability in patient responses and how this affects the reliability of the statistical analysis will be taken into account as well. More specifically, we will investigate the effect of the chronological bias [13, 23], namely a systematic temporal change in the patient outcome due to the sequential recruitment of the trial. The latter will be modeled through a linear drift in the interval $\left[-2,2\right]$, which is added to the patient response. Moreover, the case of high variability in patient responses is also considered. In such a case, the error term for the i‐th patient is generated as follows: $\epsilon_{i}=p_{i}\epsilon_{0}+\left(1-p_{i}\right)\epsilon_{1}$, where $p_{i}\sim \mathrm{Ber}\left(0.85\right)$, $\epsilon_{0}\sim N\left(0,1\right)$, and $\epsilon_{1}\sim t_{3}$, namely it is generated either from a standard normal distribution, or, with a smaller probability, from a t distribution with 3 degrees of freedom. Several settings will be explored, both with and without the presence of treatment‐by‐covariate interaction: a summary of the underlying parameters used in the simulations can be found in Table 1. In particular, in Scenario I, there is no treatment effect, whereas in Scenario II, the treatment is equally effective for all the four strata, Scenarios III and IV instead consider the case in which the treatment is effective only in three and in two strata, respectively.

**TABLE 1:** Underlying parameters for simulations.

Scenario	Δ			
	$\Delta_{1}$	$\Delta_{2}$	$\Delta_{3}$	$\Delta_{4}$
I	0	0	0	0
II	0.6	0.6	0.6	0.6
III	0	1	1	1
IV	0	0	1	1

Moreover, for each scenario, three subspecifications taking into account the presence/absence of variability in the subject outcomes are also considered: (a) no variability in patient responses, (b) presence of linear time trend, and (c) high variability in patients’ responses. In the next section, population‐based and rerandomization‐based inference will be compared in the case of pre‐ and poststratification.

## 3 Stratification in Randomization‐ Versus Population‐Based Inference

The prestratification approach makes use of a separate randomization procedure within each stratum in order to provide well‐balanced experimental groups; poststratification instead simply “ignores” such information in the allocation phase and may or may not benefit from it in the inferential phase. The first part of this work is dedicated to the comparison, in terms of the statistical power and the ability to preserve the type I error rate of the test, of the randomization versus population model approach: for each of these, the benefit of prestratifying on the covariate of interest will be investigated. In both cases, a stratified analysis will be adopted, that is, the subjects within the same stratum are compared and then the test is computed by summing the stratum‐specific tests over strata. Note that, as strata may have different sample sizes/importances, here we adopt a weighted test [1]. More specifically, within each stratum k=1,…,K, an observed test statistic $T_{\mathrm{obs},k}$ is computed so that the stratified test is obtained as

$$T_{\mathrm{obs}}=\sum_{k=1}^{K}\omega_{k}T_{\mathrm{obs}k}$$

with $\omega_{k}\in \left[0,1\right],\;k=1,\ldots ,K$ and $\sum_{k=1}^{K}\omega_{k}=1$ the stratum‐specific weights.

The measures for the rerandomization test are the simple difference in means, namely $D=\sum_{k=1}^{K}\omega_{k}d_{k}$ with $d_{k}={\bar{y}}_{\mathrm{kA}}-{\bar{y}}_{\mathrm{kB}}$ and ${\bar{y}}_{\mathrm{kj}}\;\left(j=A,B\right)$ the average response for group j in stratum k, and the linear rank test $R=\sum_{k=1}^{K}\omega_{k}\sum_{i=1}^{n_{k}}\left(a_{\mathrm{ik}}-{\bar{a}}_{k}\right)\delta_{i}$, where $\left\{a_{\mathrm{ik}}\right\}$ and ${\bar{a}}_{k}$ denote simple ranks and the mean rank in stratum k, respectively, while $n_{k}$ is the number of subjects in stratum k: the latter is the well‐known Wilcoxon rank‐sum test. For both the stratified linear rank test and the stratified difference in means, the adopted weights are proportional to the stratum‐specific sample fractions, that is, $\omega_{k}=n_{k}/n$. The p‐values for the randomization tests will be estimated by a Monte Carlo procedure. The allocation sequence is replicated L times and, each time, the test statistic $T_{l}\;\left(l=1,,,\ldots ,,,L\right)$ is computed. Thus the estimate of the two‐sided *p*‐value is obtained as the proportion of the L generated sequences with a value of the test statistic at least as extreme as $T_{\mathrm{obs}}$, namely the observed one

(1) $$\hat{p}=\frac{\sum_{l=1}^{L}I\left(|,T_{l},|,\geq ,|,T_{\mathrm{obs}},|\right)}{L}$$

where $I\left(\cdot \right)$ denotes the indicator function.

For the population model approach instead, the stratified version of the Wilcoxon test, also known as the van Elteren test, denoted as W and the stratified t‐test, denoted as t will be used. Several authors [10, 11, 12] have highlighted the fact that the usual two‐sample t‐test is generally conservative under covariate‐adaptive randomization. For this reason, the bootstrap‐based t‐test proposed by Shao, Yu, and Zhong [10] to correct the conservativeness of the traditional t‐test will be considered as well. The latter is denoted by $t_{B}$ and, as suggested by Shao, Yu, and Zhong [10], a number of B=200 bootstrap samples are used in the simulations.

Remark 1 An important distinction must be made in terms of the estimand under the two inferential approaches. The ICH E9 (R1) 2021 [24] provides a precise definition of the estimand as “a detailed description of the treatment effect that reflects the specific clinical question posed by a given clinical trial objective. It summarizes, at a population level, the potential outcomes that would occur in the same group of patients under different treatment conditions being compared.” One key difference between the two inferential frameworks lies in the selection of the population of interest. Randomization‐based inference does not require trial participants to be a random sample from a super‐population. In fact, randomization tests address a related and complementary question to that of the estimand: what is the likelihood of observing such an effect by chance alone, given a difference of this magnitude between treatments? [1] By considering what a patient's outcome would have been if they were assigned to a different treatment, randomization tests focus on the trial participants and do not seek to draw inferences about an overall treatment effect in a larger population. Instead, they provide inference specific to the trial population, relying on the effective implementation of inclusion and exclusion criteria and repetition to ensure that conclusions are applicable to the target population. More details on this distinction can be found in Uschner et al. [25].

### 3.1 Error Rates

**FIGURE 1:**
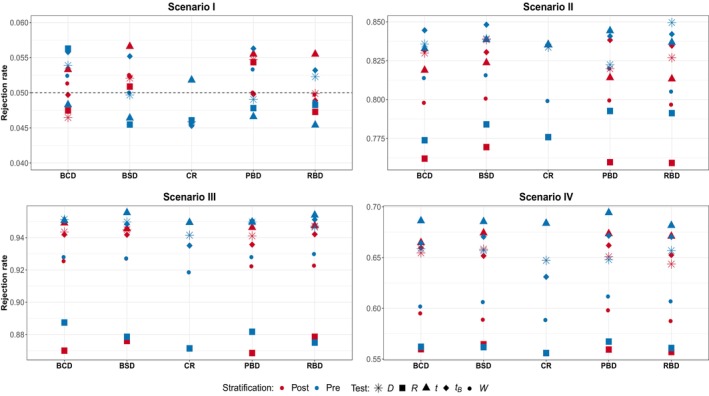
Scenario I—IV subspecification (a)—no variability in patient responses: Randomization‐based versus population‐based for pre‐ and poststratification. Considered tests: D difference in means, R linear rank test, t *t*‐test, $t_{B}$ bootstrap *t*‐test, W van Elteren test.

**FIGURE 2:**
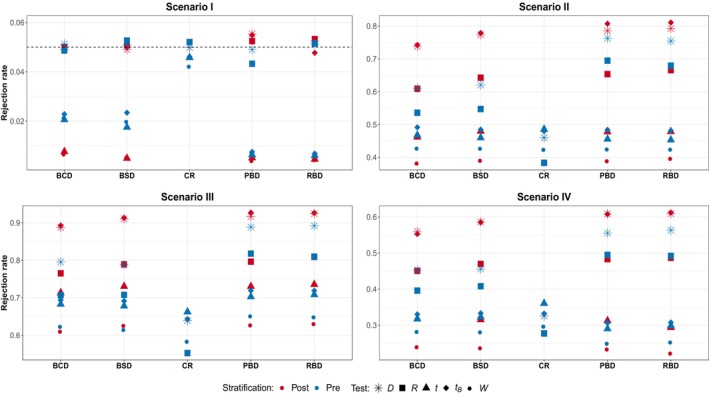
Scenario I—IV subspecification (b)—presence of linear time trend: Randomization‐based versus population‐based for pre‐ and poststratification. Considered tests: D difference in means, R linear rank test, t *t*‐test, $t_{B}$ bootstrap *t*‐test, W van Elteren test.

**FIGURE 3:**
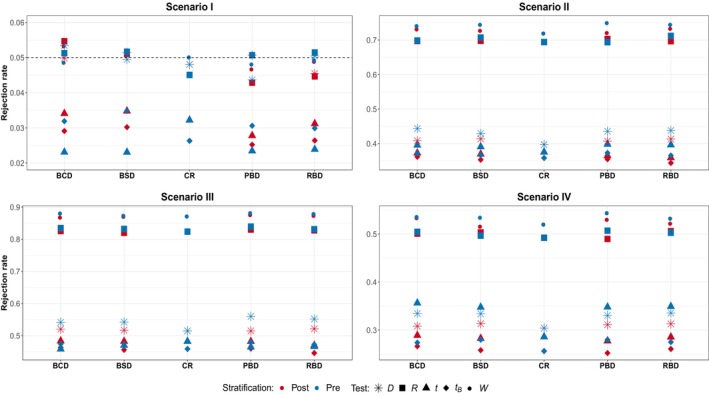
Scenario I—IV subspecification (c)—high variability in patients responses: Randomization‐based versus population‐based for pre‐ and poststratification. Considered tests: D difference in means, R linear rank test, t *t*‐test, $t_{B}$ bootstrap *t*‐test, W van Elteren test.

Figures 1, 2, 3 summarize the simulation results for Scenarios I–IV in Table 1 with pre‐ and poststratification for subspecification (a)–(c); each design is replicated 10,000 times, L is set to 20,000, and the type I error rate is set to 5%. Note that no distinction is made between pre‐ and poststratification for the CR design since it completely ignores the information provided by the covariate. Several conclusions can be drawn from these results. Starting from Figure 1 that shows the results in the case of no variability in patient responses, it can be noted that all the designs considered preserve the type I error rate without appreciable differences between pre‐ and poststratification. Looking at Scenario II instead, prestratifying seems to provide some benefit, even though of about 1%−2%, with the most notable case being W with BCD and R for block designs. The t‐tests, both the bootstrap‐based and the usual one, along with the difference in means exhibit the highest power for all the designs considered, while W and R the lowest. The bottom part of Figure 1 summarizes the results in the presence of treatment‐by‐covariate interaction, namely when the treatment is effective in three or two out of four groups. In Scenario III, it can be seen that in most cases pre‐ and poststratification provide comparable results; only for the W and R tests a small difference of about 1% can be found. Again, the tests D, t, and $t_{B}$ show the highest power while the R one the lowest. The results for Scenario IV confirm the previous pattern. Conditional on the test adopted, the influence on the power of the design and the stratification procedure adopted is not evident.

In the presence of time trend instead, Scenario I of Figure 2, only randomization‐based inference is able to maintain the prespecified type I error rate regardless of both the stratification approach and the design adopted. On the contrary, the population‐based tests seem to fail to preserve the nominal size, with the bootstrap‐based test with poststratification being the only exception, along with adopting the CR design. This is particularly true for the PBD and RBD, for which the observed values are close to 0. Here, prestratifying seems to slightly improve the ability to preserve the error rates only for the BCD and BSD. An interesting finding pertains to the behavior of the bootstrap‐based test: prestratification appears to have a detrimental effect, as the test can only preserve the type‐I error rate in the poststratification setup. Looking at the other scenarios, a clear pattern arises: prestratification seems to actually backfire. This is particularly evident for all the randomization‐based tests and the bootstrap one. Indeed, for the D test, ignoring the information provided by the covariate in the allocation phase results in an increment of power up to 15%−20% with BCD and BSD, while for $t_{B}$, the improvement is even bigger, especially for block designs. For the remaining population‐based tests instead, the differences between pre‐ and poststratification are smaller, about 5% for W with the BSD and RBD, and practically absent for the usual t‐test. Interestingly, the behavior for the rank test R greatly varies across the designs: starting from a difference of about 10% in favor of the poststratification approach for the BSD, the gap reduces for the BCD, vanishes for RBD and for the PBD the ordering is reversed, namely there is a (small) benefit in prestratifying.

In Figure 3, which summarizes the results in the presence of high variability in patients responses, no relevant differences between pre‐ and poststratification are detected. Moreover, the gap in the robustness of the two inferential approaches is confirmed. Indeed, all the randomization‐based tests preserve the nominal level, whereas for the population‐based approach, only the nonparametric W maintains the size. The t‐test and its bootstrap version suffer from a severe underestimation of the type I error rate. In all the remaining scenarios, with and without the presence of treatment‐by‐covariate interaction, it is evident that both R and W outperform all the other tests guaranteeing an improvement of more than 20% in power for all the designs. It is important to highlight that, in this setup, while all the randomization‐based tests maintain the type I error rate, only the nonparametric R is able to provide solid performance in terms of power. In the last two scenarios, a small difference of about 5% in favor of prestratification is observed for the difference in means and the t‐test.

The results so far obtained can help in providing a (partial) answer to the first question “is prestratification beneficial in terms of inferential precision?” In general, there is no clear benefit in the ability to maintain the type I error rate through prestratification. A modest improvement (less than 2%) in power is observable only in cases with no variability in patients' responses. In the presence of high variability in the outcomes, the potential benefits of prestratification are outweighed by the need to choose the appropriate test. Notably, in the presence of a chronological bias, the prestratification approach may even be deleterious, particularly when employing a randomization‐based test or the population bootstrap‐based one.

Regarding the third question: does the presence of differential treatment effects across subgroups alter the previous findings? Overall, the presence of a treatment‐by‐covariate interaction does not seem to significantly change the main results. The most notable difference between the two stratification approaches appears to be more associated with the presence of a linear time trend in the responses than with the heterogeneity among subgroups. Similar conclusions hold for both smaller (n=50) and larger (n=200) samples (see Appendix A), although as the sample size increases the differences between stratification approaches tend to vanish.

## 4 Regression Modeling

An alternative strategy for subgroup analysis exploits regression modeling in order to obtain an estimate of the covariate‐adjusted treatment effect. The test proposed by Gail, Piantadosi, and Tan [3] makes use of the residuals obtained from a model fitted with the covariate but no treatment effect, that is $E\left[Y_{i}\right]=\alpha +z_{i}^{t}\gamma$, where $z_{i}$ is the stratum indicator, namely a vector of K−1 dummies with a single nonzero entry and γ the K−1 vector of main covariate effects. Indeed, under the null hypothesis, the residuals should be evenly distributed across the treatments if there is no covariate‐adjusted treatment effect. One can then use any standard test to compare residuals in the two treatment arms, such as a difference in means, D, or ranks, namely R. The Gail et al. techniques applied only to the asymptotic distribution of the residual test under CR, but Parhat, Rosenberger, and Diao [4] demonstrate how to do this using rerandomization.

Clearly, a model including the treatment indicator, $\delta_{i}$, could also be fit, namely $E\left[Y_{i}\right]=\alpha +{\beta \delta }_{i}+z_{i}^{t}\gamma$. Here, the focus is on the ordinary least squares estimate of the covariate‐adjusted treatment effect, say $\hat{\beta }$. More specifically, after having obtained the estimate for the treatment effect of the original trial, ${\hat{\beta }}_{\mathrm{obs}}$, the allocation sequence is replicated L times and, each time, the model including the treatment indicator is estimated and its value recorded, that is ${\hat{\beta }}_{l},\left(l=1,,,\ldots ,,,L\right)$. Then the estimated two‐sided p‐value is calculated as the proportion of the L generated sequences with a value of the estimated treatment effect at least as extreme as the one observed, as described in (1). As noted in the Introduction, fitting such a model does not assume the null hypothesis that the treatment effect is zero, but the idea is consistent with the way we conduct parametric model‐fitting.

As a matter of fact, the classical population‐based approach relies on the standard t‐test on the estimated coefficient $\hat{\beta }$. In a recent work, Bugni, Canay, and Shaikh [11] proved that, in general, such a test is conservative in the sense that the limiting rejection probability under the null hypothesis could be strictly less than the nominal level. To overcome this drawback, the authors proposed an adjusted version of the t‐test with strata fixed effect that preserves the type‐I error rate under covariate‐adaptive randomization. All the techniques mentioned previously will be assessed in the following section to investigate their operating characteristics.

### 4.1 Numerical Results

In this section, we will compare the performance of five different regression modeling strategies. On the side of randomization‐based inference, we consider the approach that compares the residuals in the two groups using both differences in means and ranks, denoted by D and R, respectively. Additionally, we consider the method that uses the treatment effect as the measure of interest for the rerandomization test, indicated as B. For population‐based inference, we compare the classical techniques based on the usual t‐test on the beta coefficient, denoted as p, along with the adjusted t‐test proposed by Bugni, Canay, and Shaikh [11], denoted as $p_{\mathrm{adj}}$, aimed at preserving the type‐I error rate under covariate‐adaptive randomization.

**FIGURE 4:**
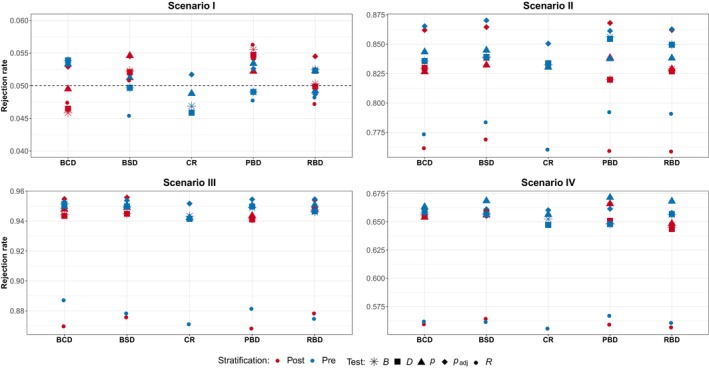
Scenario I—IV subspecification (a)—no variability in patient responses: Regression adjustment with pre‐ and poststratification. Considered approaches: D and R rerandomization‐based using difference in means and linear rank test on the residuals, respectively; B rerandomization‐based including the treatment indicator; p and $p_{\mathrm{adj}}$ population‐based using the usual t‐test and the adjusted t‐test on the beta coefficient, respectively.

The scenarios considered are the same described in Section 2; each design is replicated 10,000 times and for randomization‐based procedures we set L=20000. Figure 4 shows the simulation results for Scenario I–IV in the case of no variability in patient responses. As expected, in this set‐up the type I error rates are preserved for all the considered approaches and for all the allocation rules. The Scenario II shows the behavior of the designs considered when the treatment is equally effective in all the four groups: it is evident that the stratification approach adopted does not affect much the power of the procedure, with only a small improvement (about 1%−2%) in favor of prestratification. Moreover, the randomization test based on ranks exhibits the lowest power, while the remaining three tests seem to have similar performances, with the $p_{\mathrm{adj}}$ test showing the best values for all the considered designs. In the lower part of Figure 4 the results in the presence of treatment‐by‐covariate interaction are presented. As for the previous figures, the values of the power in each Scenario are comparable with those obtained in Section 3 highlighting that the regression‐based adjustments provide performances comparable to those obtained with a stratified analysis. This result is also in line with that obtained by Bugni, Canay, and Shaikh [11] for BCD and PBD. No discernible differences appear between the adopted stratification strategies, and apart from the test based on ranks, the others perform similarly.

**FIGURE 5:**
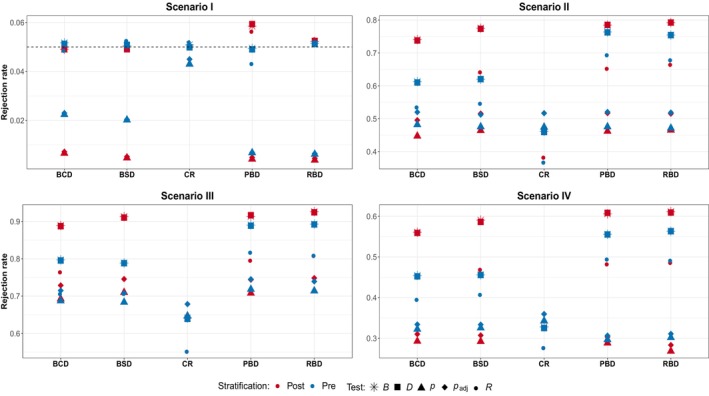
Scenario I—IV subspecification (b)—presence of linear time trend: Regression adjustment with pre‐ and poststratification. Considered approaches: D and R rerandomization‐based using difference in means and linear rank test on the residuals, respectively; B rerandomization‐based including the treatment indicator; p and $p_{\mathrm{adj}}$ population‐based using the usual t‐test and the adjusted t‐test on the beta coefficient, respectively.

This is not the case in the presence of time trend, as shown in Figure 5. All the randomization‐based procedures are able to preserve the nominal size, while the population‐based approaches suffer from a severe underestimation of the size, except under CR. There seem to be no notable differences between the two stratification approaches, only for BCD and BSD there may be a small benefit in stratifying for population‐based strategies. Looking at Scenarios II–IV, it is evident that prestratification may provide a negative benefit, especially for randomization‐based tests. In particular, for BCD and BSD stratification backfires and reduces the power up to 10%−15%, while for permuted block designs the gap is either reduced or canceled. The population‐based approach instead seems to remain neutral with respect to the stratification strategy employed.

**FIGURE 6:**
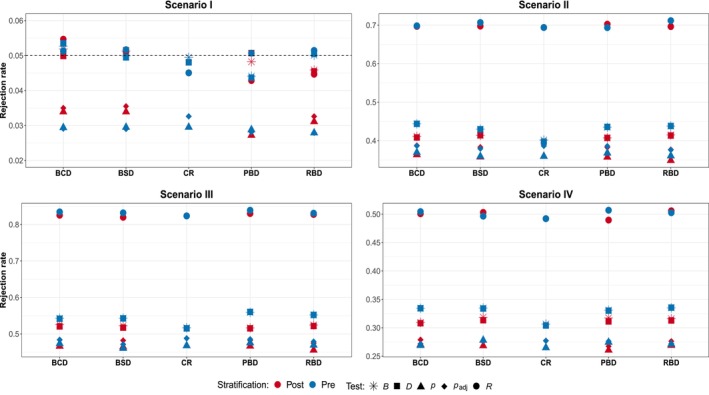
Scenario I–IV subspecification (c)—high variability in patient responses: Regression adjustment with pre‐ and poststratification. Considered approaches: D and R rerandomization‐based using difference in means and linear rank test on the residuals, respectively; B rerandomization‐based including the treatment indicator; p and $p_{\mathrm{adj}}$ population‐based using the usual t‐test and the adjusted t‐test on the beta coefficient, respectively.

In Figure 6, the results in the presence of high variability in patients' responses are summarized. The population‐based strategies do not preserve the nominal size of the test, while the randomization‐based ones confirm their robustness with respect to model misspecification. As expected, in all the other Scenarios, the rank‐based test shows the highest power, guaranteeing an improvement up to 30% compared with all the other competitors. All the other tests show similar performances with only a small difference, up to 5%, in favor of the other randomization‐based procedures compared to the population‐based ones. Interestingly, the results do not seem to be greatly affected by the stratification strategy; however, the values obtained with prestratification are generally higher, even in the presence of strong treatment‐by‐covariate interaction effects.

To summarize, what is the best rerandomization procedure in the regression‐adjustment framework? Our findings suggest that the method based on the treatment effect, namely B, provides results similar to those obtained adopting the difference in means: both methods are robust with respect to chronological bias even though in the presence of high variability their performance greatly deteriorates, with only a small improvement compared to the population‐based approach. In the latter case, as expected, the procedure based on ranks guarantees the highest power along with the preservation of the type I error rate. Similar to the previous section, the presence of heterogeneity among the subgroups does not seem to increase the benefit of stratification. In fact, even when there is treatment‐by‐covariate interaction, prestratification may backfire by considerably reducing the inferential precision.

### 4.2 Strong Versus Weak Null Hypothesis

As Neyman and Iwaszkiewicz [20] first noted, in some cases the so‐called “strong” null hypothesis of identical outcome distributions in the two treatment groups may be of no or little practical utility; especially in clinical trials [26], where the interest may lie in testing whether the first moment of the two distributions is identical rather than the distributions themselves. In such cases, the size of the rerandomization‐based test might exceed the nominal level [18, 26, 27]. In what follows, the behavior of the two inferential approaches will be compared in terms of the ability to preserve the type I error rate under the weak null hypothesis where different response distributions exist in the two competing groups, but equal first moments. More specifically, following a set‐up similar to the one described in Gail et al. [26], the error term for the i‐th patient is generated as follows: $\epsilon_{i}=\epsilon_{0}+\delta_{i}\epsilon_{1}$, where $\epsilon_{0}\sim N\left(0,1\right)$ and $\epsilon_{1}\sim N\left(0,4\right)$, namely an extra source of variability is added to the subjects in group A, that is, those with $\delta_{i}=1$. For Scenario I, Tables 2 and 3 summarize the estimated size of the tests multiplied by 1000 for stratified analysis and regression modeling; each design is simulated 10,000 times and for the rerandomization procedures the allocation sequence is replicated L=20,000 times. Note that estimates outside the interval $\left(45.7,54.3\right)$—which should include 95% of the replications by setting the size equal to 0.05—are considered to significantly differ from the nominal α=0.05 level, based on a two‐sides 0.05 level test.

**TABLE 2:** Estimated rejection probabilities (times 1000) under weak null hypothesis: Randomization‐based versus population based for pre‐ and poststratification.

	Population‐based						Re‐randomization‐based			
	Post			Pre			Post		Pre	
	t	W	$t_{B}$	t	W	$t_{B}$	D	R	D	R
BCD	54.9	60.2	48.1	57.2	63.9	54.2	52.1	63.0	52.1	60.9
BSD	54.8	57.5	50.6	53.6	59.2	54.1	56.4	62.7	45.7	56.7
CR	53.7	54.7	50.9	53.7	54.7	50.9	48.7	57.6	48.7	57.6
PBD	53.7	59.3	47.5	54.4	61.8	54.3	48.1	59.0	49.8	54.9
RBD	57.0	61.8	50.1	49.9	58.0	51.7	56.1	63.0	55.4	61.7

*Note*: Considered tests: t, *t*‐test; W, van Elteren test; $t_{B}$, bootstrap *t*‐test; D, difference in means; R, linear rank test. Estimates outside the interval $\left(45.7,54.3\right)$ differ significantly from the nominal α=0.05 level, based on a two‐sided 0.05 level test.

**TABLE 3:** Estimated rejection probabilities (times 1000) under weak null hypothesis: Randomization‐based vs. population‐based regression adjustment with pre‐ and poststratification.

	Population‐based				Re‐randomization‐based					
	Post		Pre		Post			Pre		
	p	$p_{\mathrm{adj}}$	p	$p_{\mathrm{adj}}$	D	R	B	D	R	B
BCD	50.4	58.5	55.7	60.8	52.1	63.0	51.8	52.1	60.9	51.6
BSD	51.3	59.8	50.3	56.1	56.4	62.7	55.4	45.7	56.7	45.9
CR	52.5	53.6	52.5	53.6	48.7	57.6	48.8	48.7	57.6	48.8
PBD	49.5	58.0	52.3	57.3	48.1	59.0	49.8	49.8	54.9	49.8
RBD	53.1	60.9	48.2	53.3	56.1	63.0	55.4	55.4	61.7	55.1

*Note*: Considered approaches: D and R rerandomization‐based using difference in means and linear rank test on the residuals, respectively; B rerandomization‐based including the treatment indicator; p and $p_{\mathrm{adj}}$ population‐based using the usual t‐test and the adjusted t‐test on the beta coefficient, respectively. Estimates outside the interval $\left(45.7,54.3\right)$ differ significantly from the nominal α=0.05 level, based on a two‐sided 0.05 level test.

In general, no huge departure from the nominal 0.05 levels is observed, although some clear patterns arise. From Table 2, it can be seen that, in general, both t‐based tests seem to preserve the size, especially the bootstrap‐based one which always shows values in the interval $\left(45.7,54.3\right)$. However, the nonparametric W test shows estimated sizes greater than its parametric counterpart with a maxim value of 63.9 for BCD. For all the population‐based tests the values in the prestratification case are slightly higher than those in the poststratification one. For rerandomization‐based tests instead, there is no clear distinction between the stratification strategies; the rank‐based test exhibits estimated sizes always exceeding the nominal level, while the difference in means seems to preserves the type I error rate. Different conclusions can be drawn by looking at Table 3: the population‐based technique based on the usual t‐test on the beta coefficient, namely p, generally preserves the type I error rate, while the adjusted one, $p_{\mathrm{adj}}$, tends to show higher values, with a slight inflation of the size. The randomization‐based approaches using either the difference in means D or the beta coefficient B seem to maintain the nominal level, with only few exceptions; the rank based test instead confirms the results of the previous table, its values are almost always outside the interval, with a maximum value of 63.0.

## 5 Discussion

Our results highlight several interesting points.

- Question (1): in general, prestratifying does not provide substantial benefit, on the contrary, it actually may be deleterious in many settings. This is particularly evident for randomization‐based procedures in the presence of chronological bias. This is true also for n=50 and n=200 (see the additional results in Appendix A), although in larger samples the differences between stratification strategies tend to be mitigated. In general, in the presence of high variability in the outcomes, using the appropriate nonparametric test provides more benefit than prestratification itself.
- Related to the previous point: the general validity and robustness of randomization‐based inference, especially in the presence of model misspecification is confirmed. This is particularly evident in adopting nonparametric tests in the presence of high variability in patients' responses.
- Our results regarding the behavior of the t‐test in the presence of time trend are in line with those obtained by Rosenkranz [28] and Tamm and Hilgers [23]; we further extend the analysis to the bootstrap‐based t‐test proposed by Shao, Yu, and Zhong [10], which proves to be robust only in the poststratification set‐up and to the randomization‐based tests showing that (i) in general, this approach guarantees a higher inferential accuracy, (ii) the combination of prestratification and chronological bias strongly affect the performance of BCD and BSD, (iii) RBD and PBD seem to be robust not only to the stratification approach adopted but also to the presence of a trend over time. The robustness of the block designs is not surprising, since they promote balance at intermediate points in the trial. This phenomenon has been seen in other contexts [1, 19]. Indeed, the statistical power of the permuted block designs in the case of no variability of the responses and in the presence of a linear time trend is fairly comparable.
- Question (2): the randomization‐based regression‐adjustment methods based on the residuals behave similarly to their (stratified‐analysis) counterparts with good performances in all the settings considered. The population‐based approaches confirm their validity only in the case of no variability in the patient outcomes, but greatly deteriorate otherwise, even adopting the strata‐fixed effect test proposed by Bugni, Canay, and Shaikh [11]. In general for these methods, stratifying seems to mostly have no effect. The randomization approach based on the treatment effect lies in between the previous two and shows performance similar to that obtained by adopting the difference in means in the residual‐based approach: it can handle the presence of time trend but it is strongly affected by the presence of high variability.
- Question (3): interestingly, the presence of a treatment‐by‐covariate interaction does not seem to have a disturbing effect: it clearly reduces the power of the procedures but apart from that it leaves the general picture unchanged. This is true for both stratified analysis and regression modeling.

Our grand conclusion is that prestratification does not offer advantages when poststratification and adjusted regression models can be used following the trial. This result is in line with and extends those previously obtained in the literature by considering the impact of treatment‐by‐covariate interaction and variability in patient response [6, 9, 11]. Randomization‐based inference is generally more robust than population‐based inference in the presence of some heterogeneity. Its use should not be limited these days as both stratified randomization tests and regression modeling on the residuals are easily conducted and computationally viable.

As a matter of fact, it is interesting that the CONSORT [29] document requires that the randomization procedure employed in the clinical trial be specified, but then requires nothing further. In the absence of randomization‐based inference, randomization is just a mechanism of allocation. But when randomization is treated as just a mechanism for allocation and nothing further, it becomes a barely noticeable sentence in protocols and medical journal papers. It is worth noting that Barnard recognized this phenomenon in his book review of Wald's *Sequential Analysis* in 1946: “…Professor Wald persists in an incorrect statement he has made earlier, to the effect that the classical test procedure for 2×2 tables … is not applicable to cases where the probabilities vary from trial to trial. These methods are applicable, exactly, if and only if the proper randomization procedure has been carried out–regardless of variations in probabilities” [30].

We have not discussed response‐adaptive (optimal allocation [31] or bandit [32, 33] approaches), covariate‐adaptive [1], or covariate‐adjusted response‐adaptive randomization [34] in this paper. These are more complicated procedures and each merits a detailed study on its own. With respect to response‐adaptive randomization, recent papers have shown that randomization‐based inference preserved type I error rate [27] and discussed conditional versus unconditional inference procedures [35].

While acknowledging the limitations of our analysis and the incomplete integration of randomization into the estimand framework, we hold the belief that our work can contribute to addressing questions concerning the utilization of stratification. Moreover, combining randomization‐ with population‐based tests in data analysis could be advantageous in identifying violations or misspecifications in the model assumptions.

## Conflicts of Interest

The authors declare no conflicts of interest.

## Figures and Tables

**FIGURE A1:**
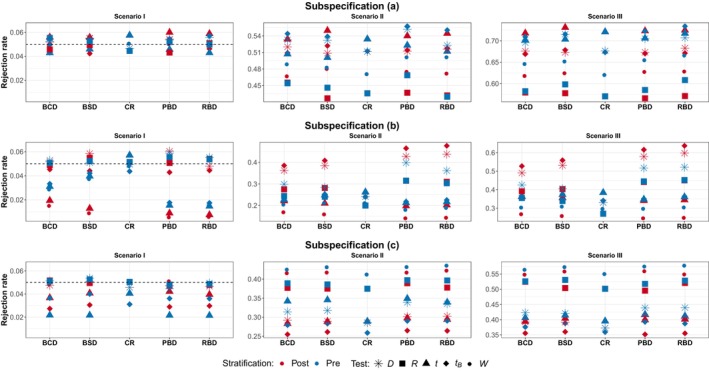
Randomization‐based versus population‐based for pre‐ and poststratification with n=50. Considered tests: D difference in means, R linear rank test, t *t*‐test, $t_{B}$ bootstrap *t*‐test, W van Elteren test.

**FIGURE A2:**
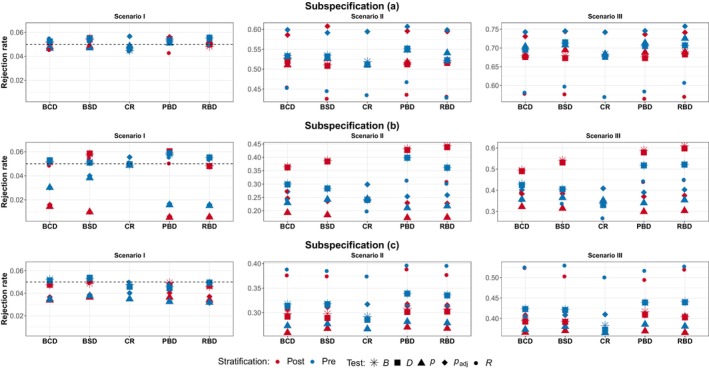
Regression adjustment for n=50 with pre‐ and poststratification. Considered approaches: D and R rerandomization‐based using difference in means and linear rank test on the residuals, respectively; B rerandomization‐based including the treatment indicator; p and $p_{\mathrm{adj}}$ population‐based using the usual t‐test and the adjusted t‐test on the beta coefficient, respectively.

**FIGURE A3:**
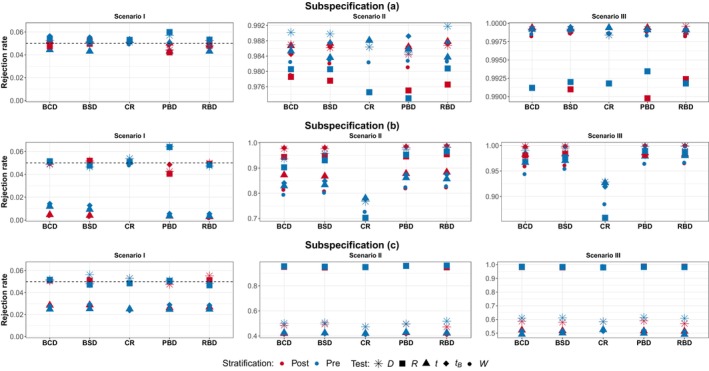
Randomization‐based versus population‐based for pre‐ and poststratification with n=200. Considered tests: D difference in means, R linear rank test, t *t*‐test, $t_{B}$ bootstrap *t*‐test, W van Elteren test.

**FIGURE A4:**
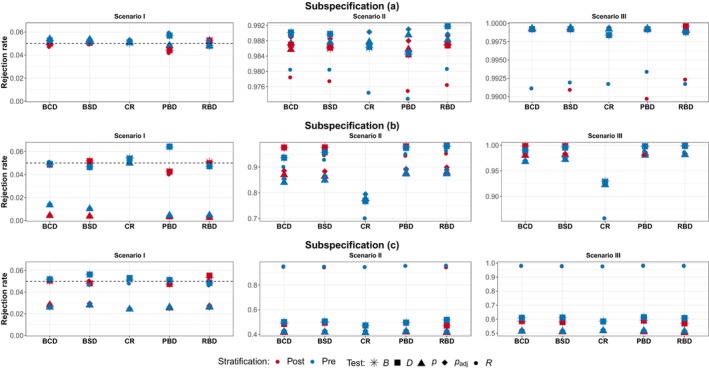
Regression adjustment for n=200 with pre‐ and poststratification. Considered approaches: D and R rerandomization‐based using difference in means and linear rank test on the residuals, respectively; B rerandomization‐based including the treatment indicator; p and $p_{\mathrm{adj}}$ population‐based using the usual t‐test and the adjusted t‐test on the beta coefficient, respectively.

## Data Availability

Data sharing is not applicable to this article as no new data were created or analyzed in this study.

## Appendix A

### A.1

Additional results for n=50 and n=200 (Figures A1, A2, A3, A4).
